# Years of Life Lost Due to Premature Death and Their Trends in People With Selected Neurological Disorders in Shanghai, China, 1995–2018: A Population-Based Study

**DOI:** 10.3389/fneur.2021.625042

**Published:** 2021-03-05

**Authors:** Zheng Luo, Huihui Lv, Yichen Chen, Xiaoyun Xu, Kangyong Liu, Xiaopan Li, Yang Deng, Yi Zhou

**Affiliations:** ^1^Department of Neurology, Shanghai University of Medicine & Health Sciences Affiliated Zhoupu Hospital, Shanghai, China; ^2^Department of Neurology, Yueyang Hospital of Integrated Traditional Chinese and Western Medicine, Shanghai University of Traditional Chinese Medicine, Shanghai, China; ^3^Center for Disease Control and Prevention of Pudong New Area, Shanghai, China; ^4^Office of Scientific Research and Information Management, Fudan University Pudong Institute of Preventive Medicine, Shanghai, China; ^5^School of Public Health, Shandong First Medical University & Shandong Academy of Medical Sciences, Tai'an, China

**Keywords:** neurological disorder, mortality, years of life lost, trend analysis, decomposition method

## Abstract

**Background:** Neurological disorders are the leading cause of long-term disability and the second leading cause of death in the world. We aimed to characterize the long-term trends in mortality and disease burden of selected neurological disorders and quantitatively analyze the contributions of demographic and non-demographic factors on the mortality of selected neurological disorders in Shanghai, China, 1995–2018.

**Methods:** Mortality data were derived from the Vital Statistics System of Pudong New Area, Shanghai, China, during 1995–2018. Temporal trends for the mortality rates and burden of selected neurological disorders were analyzed by Joinpoint Regression Program. Years of life lost (YLL) was used to analyze the burden of disease. The increasing mortality rates related to demographic and non-demographic factors were estimated by the decomposition method.

**Results:** A total of 4432 deaths from selected neurological disorders occurred during 1995–2018, accounting for 0.98% of total deaths. The crude mortality rates (CMR) and age-standardized mortality rates (ASMRW) of neurological disorders were 7.14/10^5^ person–years and 4.08/10^5^ person–years, respectively. Extrapyramidal and movement disorders, other degenerative diseases of the nervous system, and episodic and paroxysmal disorders were the three leading causes of mortality and YLL of selected neurological disorders. The CMR, ASMRW, and rate of YLL for deaths from selected neurological disorders showed significantly increasing trends in males, females, and the total population during 1995–2018 (all *P* < 0.001). The contribution rates of increased values of CMR related to demographic factors were more evident than non-demographic factors.

**Conclusion:** The mortality rate and rate of YLL for death from selected neurological disorders increased significantly during 1995–2018 in Pudong New Area, Shanghai. The demographic factors, particularly aging, might be related to an increase in the mortality of neurological disorders. More effective prevention strategies are needed to prevent the aging-related death and burden from neurological disorders in the future.

## Introduction

Globally, the burden of neurological disorders has increased substantially in the past two to three decades. Neurological disorders ranked as the leading cause of disability-adjusted life-years (DALYs, 276 million) and the second leading cause of death (9.0 million, comprising of 16.5% of global deaths) in 2016 (1). The increasing burden is possibly attributed to rapid demographic and epidemiological transitions such as the explosive population growth, aging, and unhealthy lifestyles (2). The dramatic demographic and socioeconomic changes result in more people reaching ages where neurological disorders are most prevalent worldwide, especially in the low- and middle-income countries (LMICs). For example, the life expectancy at birth increased from 68 years to 76 years in China, and from 58 years to 66 years in India during 1990–2013 (3).

These factors are usually categorized as demographic factors such as age, gender, district of residence, educational status, ethnicity, and family relationships. Population aging is recognized as the most important demographic factor for most neurological disorders. The prevalence of most neurological disorders increases gradually with aging, and aging can regulate the effects of non-demographic factors, clinical manifestation, and natural history of these disorders (4). Non-demographic factors are referred to factors arising from circumstances beyond the individual's control, e.g., economic resources, access to healthcare, development of medical technology, changing of living environment, and improvement of residents' health awareness (5). However, the contributions of demographic and non-demographic factors to the burden of neurological disorders have not been quantitatively demonstrated. China is one of the largest LMICs. Now, China is aging at an extraordinary speed and has the largest quantity of elderly persons in the world. Thus, the burden of neurological disorders should be considered a public health priority in China.

The Global Burden of Disease (GBD) 2015 Neurological Disorders Collaborator Group reported that age-standardized incidence, mortality, and prevalence rates of many neurological disorders decreased in many countries from 1990 to 2015, whereas the absolute number of patients dying or remaining disable from neurological disorders increased globally (2). Previous epidemiological studies on neurological disorders have used incidence, mortality, and prevalence rate to describe the epidemiology of neurological disorders, but these indicators do not reflect the complete burden and impact on public health and society (6). Years of life lost (YLL), a measure of premature mortality, is usually used to better quantify the burden on public health from a particular cause of death (7). Regular GBD reports on the incidence, prevalence, mortality, and YLL of neurological disorders are important for evidence-based health-care planning, priority setting, and resource allocation (1, 2). However, these indicators had large geographic variations. It is needed to estimate the regional morality and burden of neurological disorders, which can help to guide health systems to reduce the burden from neurological disorders in local areas. In this population-based study, we analyzed the long-term trends in the mortality and burden of selected neurological disorders in Pudong New Area, a typical sample of Shanghai, during 1995–2018, and characterized the impacts of demographic and non-demographic factors on the mortality, which may help in optimizing control strategies for these disorders in other areas, especially in LMICs.

## Materials and Methods

### Data Source

The death data of registered permanent residents with neurological disorders from 1995 to 2018 were derived from the mortality registration system of Pudong New Area. The Mortality Registration System of Pudong New Area, covers medical institutions of all levels and data is checked against local population registry on a monthly basis. Periodic evaluations, data cleaning, and compilation have been done at both the county and provincial levels according to standard guidelines (8). The infant death reporting system has been established in local hospitals since 1974. The funeral and cremation system has been implemented throughout the city since 1980s (9). All these measures ensure the completeness of the registration system to the maximum extent. The detailed data including age, gender, date, and cause of death were collected. The population data were provided by the Public Security Bureau of Pudong New Area. According to the standard guidelines, periodic evaluations, data cleaning, and compilation are performed to ensure the completeness of the registration system (9).

The detailed working procedure for mortality registration in Pudong New Area is described in the guidelines for surveillance in the Diseases Surveillance Points System (8). Data for 1995–2001 and 2002–2018 were coded based on the International Classification of Diseases, 9th and 10th Revision (ICD-9 and ICD-10), respectively. All the data were then analyzed according to the Chinese Classification of Diseases (CCD), due to previous use in Shanghai's annual report and assisted to bridge ICD-9 and ICD-10. All causes of death were coded by rigorously trained neurologists, and each record was further verified by local Center for Disease Control and Prevention (CDC). Review of medical records, reports from family members, or police records were carried out if there were any discrepancies.

We used the underlying cause of death (referred to as recorded underlying cause), which was a commonly used indicator in the cause-of-death statistics. The underlying cause of death was determined through a rigorous medical certificate, which was completed by a medical examiner, coroner, or other certifier, and it elicited relevant information on the nature of the death. Then, we classified deaths based on the underlying cause of death information into categories by causes using ICD-10 codes for death. The causes of death coded as G00–G99 according to ICD-10 codes were selected in this study, whereas stroke was not included because it was categorized into the diseases of the circulatory system (I00–I99) in the ICD-10 coding system. The causes of death from diseases of the nervous system (G00–G99) including inflammatory diseases of the central nervous system (G00–G09), systemic atrophies primarily affecting the central nervous system (G10–G13), extrapyramidal and movement disorders (G20–G26), other degenerative diseases of the nervous system (G30–G32), demyelinating diseases of the central nervous system (G35–G37), episodic and paroxysmal disorders (G40–G47), nerve, nerve root, and nerve plexus disorders (G50–G59), polyneuropathies and other disorders of the peripheral nervous system (G60–G64), diseases of nerve junction and muscle (G70–G73), cerebral palsy and other paralytic syndromes (G80–G83), and other disorders of the nervous system (G90–G99) according to ICD-10 were analyzed. The study was performed in accordance with the 2000 Declaration of Helsinki and was approved by the ethics committee of Shanghai Pudong New Area Center for Disease Control and Prevention.

### Statistical Analyses

The crude mortality rates (CMR) and age-standardized mortality rates (ASMRW) by Segi's world standard population of selected neurological disorders were calculated and shown as per 100,000 (/10^5^). The CMR and ASMRW between genders were compared by the Poisson approximation method and Mantel–Haenszel test, respectively. Age-specific CMR were calculated in the age groups of 0–4 years, 5–14 years, 15–29 years, 30–44 years, 45–59 years, 60–69 years, 70–79 years, and ≥80 years.

YLL was used to analyze the burden of selected neurological disorders according to the method proposed by Murray and Lopez (10). YLL was estimated using the following formula adopted by World Health Organization (WHO) in the GBD study (11):

(1) $$\begin{array}{l} YLL=KCe^{\text{ra}}/{\left(r+\beta \right)}^{2}\{e^{-(\text{r}+\beta )(\text{L}+\text{a})}[-(r+\beta )(L+a)-1]\\ \;\;\;\;\;\;\;\;\;\;\;-e{-}^{(r+\beta )a}[-(r+\beta )a-1]\}+(1-K)/r^{*}(1-e^{-\text{rL}}) \end{array}$$

where *K* refers to the use of age-specific weight (using age-weight, applied 1; not using age-weight, applied 0), C denotes the age-weighting constant (0.1658), *r* represents the discount rate (0.03), β is a parameter of age-weighting function (0.04), L indicates the standard life expectancy at the age of death according to the standard reference life table for the GBD study (80 years in males and 82.5 years in females), a refers to the average age at death, and e represents the Napier's constant.

Temporal trends in CMR, ASMRW, and the increasing rate of YLL were calculated using Joinpoint Regression Program 4.3.1.0 (downloaded from the website of the National Cancer Institute, MD, USA) and expressed as an annual percent change (APC) with corresponding 95% confidence interval (CI). The *Z* test was used to evaluate whether the APC was statistically different from zero. The increasing mortality rates of each period in 3 years from 1998 to 2018, compared with the data of the first period (1995–1997), related to demographic and non-demographic factors were estimated by the decomposition method, in which mortality rates were calculated and compared for each 5-year age group, from 0–4 to 85+ years (12). All statistical analyses were conducted using SPSS 21.0 (SPSS, Inc., Chicago, IL) and R (version 3.4.3). *P-*value of < 0.05 was considered as statistically significant.

## Results

### Baseline Characteristics of Death From Neurological Disorders

From 1995 to 2018, all registered permanent residents in Pudong New Area, with a total of 62,071,780 person–years, were enrolled in this study. There were 4,432 deaths from selected neurological disorders, accounting for 0.98% of all-cause deaths in the same period. The median age at death from selected neurological disorders was 74.04 years old, and the average age at death was 68.41 ± 20.26 years old. A total of 2,411 men and 2,021 women died of selected neurological disorders. The CMR and ASMRW of selected neurological disorders were 7.14/10^5^ person–years and 4.08/10^5^ person–year, respectively. The CMR and ASMRW were 7.78/10^5^ person–years and 4.93/10^5^ person–years in males, while the corresponding rates were 6.50/10^5^ person–years and 3.32/10^5^ person–years in females. The CMR and ASMRW were higher in males than in females (all *P* < 0.05) (Table 1).

**Table 1:** Baseline characteristics of deaths and burden in different genders and types of selected neurological disorders during 1995–2018.

**Characteristic**	**Deaths** **(*n*, %)**	**Age at death** **(Mean ± SD)**	**Age at death** **(Median)**	**CMR** **(/10**^**5**^**)**	**ASMRW** **(/10**^**5**^**)**	**YLL** **(years)**	**YLL rate** **(/10**^**5**^**)**
**Gender**							
Male	2411 (54.40)	66.32 ± 20.36	72.04	7.78	4.93	29,827.57	96.29
Female	2021 (45.60)	70.90 ± 19.85	77.07	6.50	3.32	23,784.38	76.49
**Type**							
Inflammatory diseases of the central nervous system (G00–G09)	181 (4.08)	51.81 ± 23.47	56.55	0.29	0.26	3285.13	5.29
Systemic atrophies primarily affecting the central nervous system (G10–G13)	328 (7.40)	59.77 ± 15.87	62.25	0.53	0.35	5207.09	8.39
Extrapyramidal and movement disorders (G20–G26)	1513 (34.14)	77.49 ± 9.38	78.83	2.44	1.00	12,999.36	20.94
Parkinson's disease (G20–G22)	1495 (33.73)	77.67 ± 9.21	78.93	2.41	0.91	12,727.60	20.50
Other degenerative diseases of the nervous system (G30–G32)	1196 (26.99)	77.58 ± 12.99	80.49	1.93	0.82	10,540.65	16.98
Alzheimer's disease (G30)	256 (5.78)	82.44 ± 9.43	83.85	0.41	0.16	1811.26	2.92
Demyelinating diseases of the central nervous system (G35–G37)	33 (0.74)	59.62± 15.35	62.73	0.05	0.03	544.31	0.88
Episodic and paroxysmal disorders (G40–G47)	546 (12.32)	51.54 ± 22.81	51.39	0.88	0.70	10,095.72	16.26
Epilepsy (G40–G41)	527 (11.89)	50.85 ± 22.71	50.71	0.69	0.67	9881.30	15.92
Nerve, nerve root, and nerve plexus disorders (G50–G59)	26 (0.59)	63.10 ± 17.48	71.13	0.04	0.03	368.95	0.59
Polyneuropathies and other disorders of the peripheral nervous system (G60–G64)	33 (0.74)	63.24 ± 13.40	63.86	0.05	0.03	487.87	0.79
Diseases of nerve junction and muscle (G70–G73)	205 (4.63)	51.02 ± 23.25	54.69	0.33	0.29	3760.91	6.06
Cerebral palsy and other paralytic syndromes (G80–G83)	159 (3.59)	40.92 ± 28.34	34.24	0.26	0.32	3345.32	5.39
Other disorders of the nervous system (G90–G99)	212 (4.78)	62.95 ± 23.17	69.46	0.34	0.24	2976.63	4.80
Total	4432 (100.00)	68.41 ± 20.26	74.04	7.14	4.08	53,611.95	86.37

*ASMRW, age-standardized mortality rate by Segi's world standard population; CMR, crude mortality rate; YLL years of life lost*.

The rank of causes of death from selected neurological disorders in males or females were consistent with that in total population, and the top three were extrapyramidal and movement disorders (G20–G26), other degenerative diseases of the nervous system (G30–G32), and episodic and paroxysmal disorders (G40–G47), respectively. Parkinson's disease (PD) (G20–G22) accounted for 98.81% of G20–G26, Alzheimer's disease (AD) (G30) accounted for 21.40% of G30–G32, and epilepsy (G40–G41) accounted for 96.52% of G40–G47. The CMR, ASMRW, the average age and median age at death in different genders, and types of selected neurological disorders are shown in Table 1.

### Age-Specific Mortality of Neurological Disorders

The number of the elderly over 60 years old who died of selected neurological disorders was 3,331, accounting for 75.16% of the total population. The mortality rates in the age groups of 0–4 years, 5–14 years, 15–29 years, 30–44 years, 45–59 years, 60–69 years, 70–79 years, and ≥80 years were 2.91/10^5^ person–years, 1.11/10^5^ person–years, 1.70/10^5^ person–years, 1.63/10^5^ person–years, 3.56/10^5^ person–years, 10.01/10^5^ person–years, 28.51/10^5^ person–years, and 73.17/10^5^ person–years, respectively (Table 2).

**Table 2:** Age-specific mortality and burden of selected neurological disorders during 1995–2018.

**Age group (years)**	**Deaths (*N*)**	**Proportion (%)**	**CMR (/10^**5**^)**	**YLL (years)**	**YLL rate (/10^**5**^)**
0–4	65	1.46	2.91	1,938.44	86.74
5–14	57	1.29	1.11	1,700.96	33.06
15–29	192	4.33	1.70	5,282.82	46.87
30–44	249	5.62	1.63	6,021.68	39.50
45–59	538	12.14	3.56	10,359.99	68.66
60–69	706	15.93	10.01	10,045.83	142.29
70–79	1,139	25.70	28.51	10,563.29	264.59
80+	1,486	33.53	73.17	7,698.95	378.74
Total	4,432	100.00	7.14	53,611.95	86.37

ASMRW, age-standardized mortality rate by Segi's world standard population; CMR, crude mortality rate; YLL, years of life lost.

Cerebral palsy and other paralytic syndromes (G80–G83) were the leading cause of death in the age group of 0–14 years, while episodic and paroxysmal disorders (G40–G47) were the leading cause of death in the age groups of 15–44 years and 45–64 years. Furthermore, the proportions of top three causes of death in the age group of 45–64 years were very similar, such as episodic and paroxysmal disorders (G40–G47), other degenerative diseases of the nervous system (G30–G32), and systemic atrophies primarily affecting the central nervous system (G10–G13), respectively. Extrapyramidal and movement disorders (G20–G26) were the leading cause of death among residents aged over 65 years. In addition, other degenerative diseases of the nervous system (G30–G32) were the main cause of death among residents aged over 80 years. The rank of cause of death and the proportion in each age group are presented in Supplementary Figure 1.

### Burden of Premature Death From Neurological Disorders

During 1995–2018, the YLL due to premature death from selected neurological disorders was 53,611.95 years, and the rate of YLL was 86.37/10^5^. YLL and rates of YLL in males (29,827.57 years, 96.29/10^5^) were higher than those in females (23,784.38 years, 76.49/10^5^). For causes of mortality, YLL and rate of YLL due to extrapyramidal and movement disorders (G20–G26) were highest (12,999.36 years, 20.94/10^5^), followed by other degenerative diseases of the nervous system (G30–G32) (10,540.65 years, 16.98/10^5^) and episodic and paroxysmal disorders (G40–G47) (10,095.72 years, 16.26/10^5^) (Table 1). In terms of age, the top three in YLL were in the age groups of 70–79 years, 45–59 years, and 60–69 years, which were 10,563.29 years, 10,359.99 years, and 10,045.83 years, respectively. The top three age groups in the rates of YLL were 80+ years, 70–79 years, and 60–69 years, which were 378.71/10^5^, 264.59/10^5^, and 142.29/10^5^, respectively (Table 2).

### Trends of Mortality and Burden of Neurological Disorders

**Figure 1:**
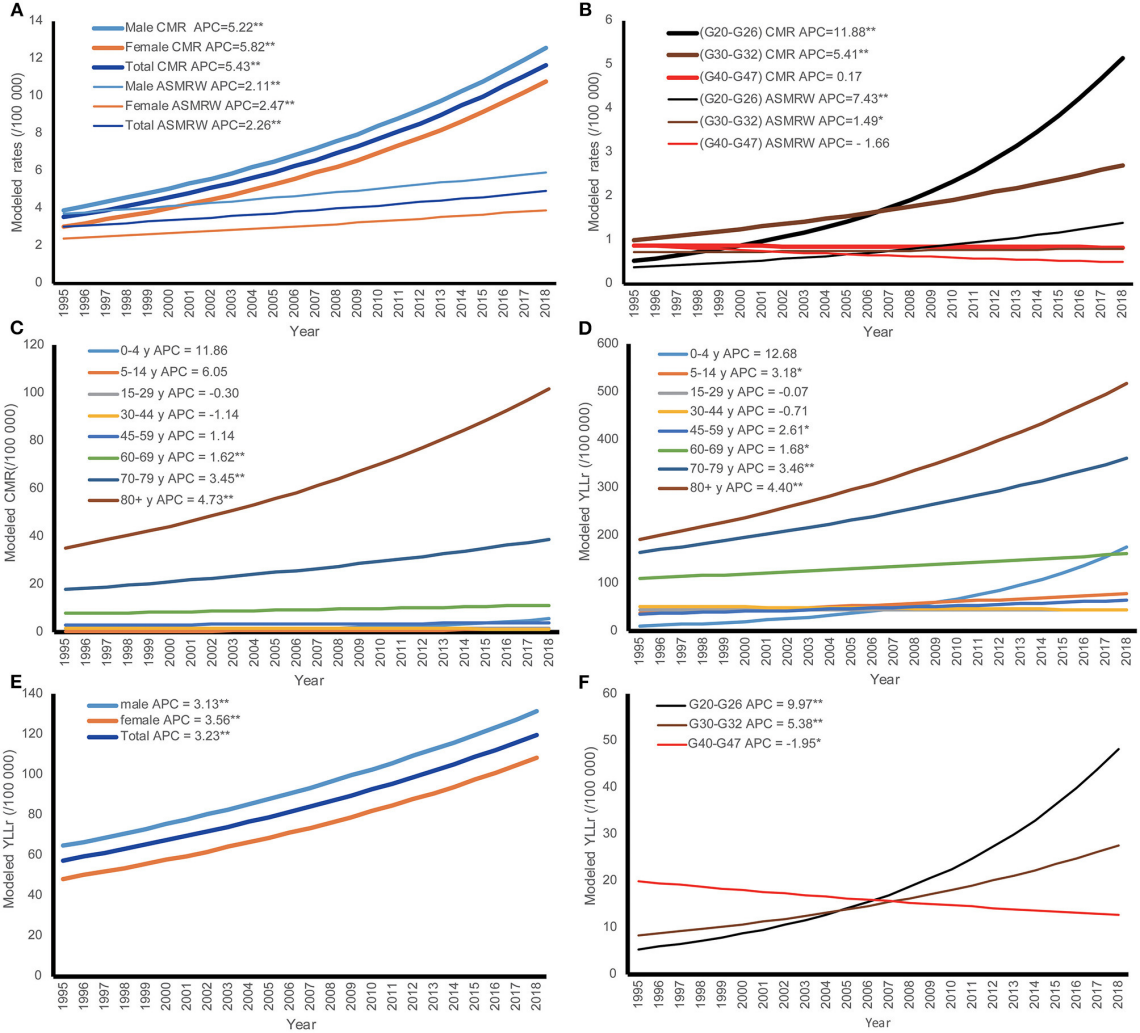
The trends in CMR, ASMRW, and YLL of persons with underlying cause of death from selected neurological disorders in genders, major pathology types, and age groups in Pudong New Area, Shanghai, China, 1995–2018. **(A)** Trends in CMR and ASMRW in genders; **(B)** trends in CMR and ASMRW in major pathology types; **(C)** trends in CMR among different age groups; **(D)** trends in YLL among different age groups; **(E)** trends in YLL in genders; **(F)** trends in YLL in major pathology types. ASMRW, age-standardized mortality rate by Segi's world standard population (per 100,000); CMR, crude mortality rate (per 100,000); G20–G26, extrapyramidal and movement disorders; G30–G32, other degenerative diseases of the nervous system; G40–G47, episodic and paroxysmal disorders. **P < 0.001; *P < 0.05.

The temporal trends in CMR, ASMRW, and rate of YLL are expressed based on the modeled CMR, ASMRW, and rate of YLL and shown in Figure 1. The CMR, ASMRW, and rate of YLL for deaths from selected neurological disorders showed significantly increasing trends in males, females, and the total population during 1995–2018 (all *P* < 0.001) (Figures 1A,E and Table 3). For the major types of selected neurological disorders, the CMR of extrapyramidal and movement disorders (G20–G26) increased by 11.88% (95% CI = 10.79–12.98%, *P* < 0.001) per year during the study period. Furthermore, ASMRW and YLL rate of extrapyramidal and movement disorders (G20–G26) increased significantly. There were significant upward trends in the CMR, ASMRW, and YLL rate of other degenerative diseases of the nervous system (G30–G32). The YLL rate of episodic and paroxysmal disorders (G40–G47) decreased with an APC of −1.95% (95% CI = −3.51 to −0.37%, *P* < 0.05) per year from 1995 to 2018, while the CMR and ASMRW remained stable (Figures 1B,F and Table 3).

**Table 3:** Trends of CMR, ASMRW, and YLL rate of selected neurological disorders in genders and major types during 1995–2018.

**Characteristics**	**APC (95% CI) (%)**		
	**CMR**	**ASMRW**	**YLL rate**
**Gender**			
Male	5.22 (4.10, 6.36)^**^	2.11 (1.08, 3.15)^**^	3.13 (1.88, 4.40)^**^
Female	5.82 (5.12, 6.52)^**^	2.47 (1.51, 3.43)^**^	3.56 (2.74, 4.38)^**^
**Type**			
Extrapyramidal and movement disorders (G20–G26)	11.88 (10.79, 12.98)^**^	7.43 (6.33, 8.54)^**^	9.97 (8.67, 11.28)^**^
Other degenerative diseases of the nervous system (G30–G32)	5.41 (4.04, 6.81)^**^	1.49 (0.04, 2.96)^*^	5.38 (3.61, 7.18)^**^
Episodic and paroxysmal disorders (G40–G47)	0.17 (−1.20, 1.57)	−1.66 (−3.53, 0.24)	−1.95 (−3.51, −0.37)^*^
Total	5.43 (4.86, 5.98)^**^	2.26 (1.72, 2.81)^**^	3.23 (2.63, 3.84)^**^

APC, annual percent change; ASMRW, age-standardized mortality rate by Segi's world standard population; CMR, crude mortality rate; CI, confidence interval; YLL, years of life lost.

* P < 0.05.

** *P < 0.001*.

In terms of age-specific mortality and burden, CMR and ASMRW of the total population had showed upward trends from 1995 to 2018. The increasing trends of CMR were also seen in the age groups of 60–69 years, 70–79 years, and 80+ years. The rates of YLL increased by 3.23% (95% CI = 2.63–3.84%, *P* < 0.001) per year in the total population, 3.18% (95% CI = 0.30–6.14%, *P* < 0.05) per year in the age group of 5–14 years, 2.61% (95% CI = 0.98–4.27%, *P* < 0.05) per year in the age group of 45–59 years, 1.68% (95% CI = 0.65–2.72%, *P* < 0.05) per year in the age group of 60–69 years, 3.46% (95% CI = 2.41–4.52%, *P* < 0.001) per year in the age group of 70–79 years, and 4.40% (95% CI = 3.25–5.56%, *P* < 0.001) per year in the age group of 80+ years, respectively (Figures 1C,D and Table 4).

**Table 4:** Trends of CMR, ASMRW, and YLL rate of selected neurological disorders in different age groups during 1995–2018.

**Age Group(years)**	**APC (95% CI) (%)**		
	**CMR**	**ASMRW**	**YLL rate**
0–4	11.86 (−4.14, 30.54)		12.68 (−4.74, 33.29)
5–14	6.05 (−8.74, 23.23)		3.18 (0.30, 6.14)^*^
15–29	−0.30 (−2.23, 1.67)	/	−0.07 (−2.17, 2.09)
30–44	−1.14 (−3.33, 1.09)	/	−0.71 (−2.71, 1.34)
45–59	1.14 (−0.22, 2.53)	/	2.61 (0.98, 4.27)^*^
60–69	1.62 (0.57, 2.68)^*^	/	1.68 (0.65, 2.72)^*^
70–79	3.45 (2.47, 4.44)^**^	/	3.46 (2.41, 4.52)^**^
80+	4.73 (3.60, 5.87)^**^	/	4.40 (3.25, 5.56)^**^
Total	5.43 (4.86, 5.98)^**^	2.26 (1.72, 2.81)^**^	3.23 (2.63, 3.84)^**^

APC, annual percent change; ASMRW, age-standardized mortality rate by Segi's world standard population; CI, confidence interval; CMR, crude mortality rate; YLL, years of life lost.

* P < 0.05.

** P < 0.001.

### Quantitatively Impacts of Demographic and Non-demographic Factors on Increased Rates in CMR

**Figure 2:**
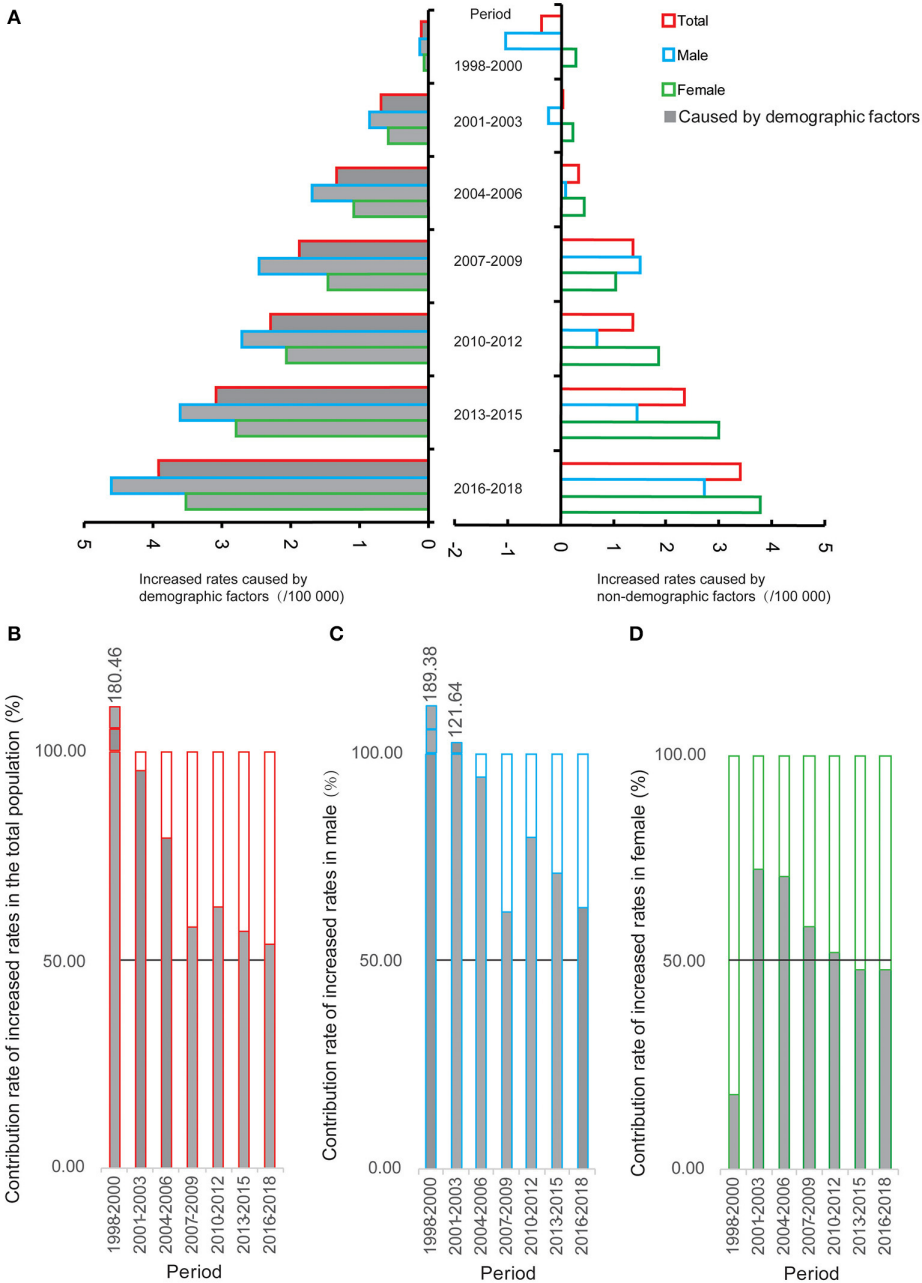
The increased rates related to demographic and non-demographic factors and their proportion during the period from 1998 to 2018 compared with the crude mortality rate of selected neurological disorders during 1995–1997 in Pudong New Area, Shanghai, China. **(A)** The increased rates; **(B)** the contribution rate of increased rates in the total population; **(C)** the contribution rate of increased rates in males; **(D)** the contribution rate of increased rates in females.

The trends of increased rates in CMR related to non-demographic and demographic factors are shown in Figure 2A. Based on the CMR of selected neurological disorders in 1995–1997, there was a significant increasing trend in the increased rate related to non-demographic factors in the total population, with an APC of 25.51% (95% CI = 12.63–39.87%, *P* < 0.001) from 1998 to 2018, and a significant upward trend was also observed in the increased rate related to demographic factors [APC (95% CI) = 70.50% (25.13–132.31%), *P* = 0.007]. In males, the increased rate caused by non-demographic factors related to 36.51% (95% CI = 27.83–45.79%, *P* < 0.001) during 1998–2018, and the rate related to demographic factors increased by 66.06% (95% CI = 22.52–125.08%, *P* = 0.008). In females, the increased rate related to non-demographic factors showed an upward trend during 1998–2018, with an APC of 67.09% (95% CI = 44.09–93.75%, *P* < 0.001), and the rate related to demographic factors also increased [APC (95% CI) = 76.24% (27.39–143.83%), *P* = 0.007].

The proportion of increased values of CMR related to non-demographic factors and demographic factors are shown in Figures 2B–D and Supplementary Table 1. Compared to the CMR during 1995–1997, the contribution rates of increased values of CMR related to demographic factors were over 50% in the total population from 1998 to 2018. Interestingly, the impact of demographic factors on the CMR during 1998–2018 was more evident in males than in females.

## Discussion

As the largest metropolis in China, Shanghai is one of the earliest cities to enter the upper-middle-income region and the aging society. Shanghai is the forerunner of demographic and socioeconomic development in China. What is happening in Shanghai will be repeated in many cities in China and other LMICs over the next 20 years (13). Therefore, Shanghai is one of the most suitable models to characterize the impacts of demographic and socioeconomic changes on the death of neurological disorders. We chose Pudong New Area as a research area for several reasons. First, Pudong New Area is the largest district of Shanghai with a permanent resident population of 3.0 million, accounting for over 20% of the total permanent resident population in Shanghai in 2018. Second, since the founding of Pudong New Area in 1993, it has become the most populous district undergoing the rapid urbanization. In addition, the immigration of non-native population and aging of natives have caused tremendous changes in the demographic structure. It is regarded as the microcosm of China's reformation and a good representative of Shanghai (14). Third, Pudong New Area has established a reliable mortality registration system covering 100% of permanent resident population, which provides good quality data to analyze the mortality of diseases.

China accounts for approximately 21.6% of the global DALYs from neurological disorders, so understanding the mortality and burden attributed to neurological disorders is essential (1). In this population-based study, we found that PD, epilepsy, and AD were top three leading causes of death from selected neurological disorders, and CMR, ASMRW, and YLL rates of patients with PD and AD increased significantly from 1995 to 2018. AD and PD are common neurodegenerative disorders. Aging is the most important risk factor for these two disorders, and other risk factors include genetic predisposition, unhealthy lifestyle behaviors, and various environmental agents (15). One-tenth of the elderly aged above 65 years have AD, and PD affects about 1% of individuals aged above 60 years and 5% of individuals over 85 years (16). Aging is an evolutionarily conserved natural process that involves dysregulation of various signaling pathways, such as oxidative stress, mitochondrial dysfunction, autophagy, and neuroinflammation, which are also involved in neurodegenerative disorders (17). Emerging evidences demonstrate that unhealthy lifestyle behaviors such as lack of physical exercise, high intake of saturated fat, and anxiety or depression are positively associated with the development of PD and AD (18, 19). For example, western lifestyle, which is characterized by high intake of red and processed meat, low vegetables and fruits intake, and physical inactivity, has been regarded as an established risk factor for AD and PD development (20). After being designated as an Open Economic Zone, the economy of Pudong New Area has achieved rapid development and more residents have adopted the western lifestyles (13). According to a dietary survey in urban Shanghai, the proportion of intake of red meat increased from 9% in 1982 to 26% in 2002, whereas the rate of cereal intake declined from 65 to 40% (21). Environmental factors also play an important role in the etiology of AD and PD. Some industrial pollutants and chemicals tied to industrialization including volatile solvents, heavy metals, herbicides, and pesticides may contribute to the increasing incidence of PD and AD (22–24). Except for those unmodifiable risk factors that include age, sex, race, and genetics, these evidences suggest that AD and PD should be prevented by developing healthy lifestyles such as increasing physical activity and consuming more vegetables and fruits, and decreasing the exposure to industrial pollutants.

We also observed noteworthy differences in mortality and YLL between age groups. Among the individuals aged 15–44 years, episodic and paroxysmal disorders (G40–G47) were the leading cause of death, while extrapyramidal and movement disorders (G20–G26) and other degenerative diseases of the nervous system (G30–G32) were the most significant contributors to the mortality of individuals aged over 65 years. YLL was highest in the age group of 70–79 years, followed by those in the age groups of 45–59 years and 60–69 years. Moreover, the CMR and YLL showed increasing trends in the age groups of 60–69 years, 70–79 years, and 80+ years, respectively. The underlying reason for these phenomena is the aggravating trend of aging population. Shanghai is the first city to enter the aging society, and also the most aging metropolis in China. In 2008, Pudong New Area entered the aging society, and the proportion of people aged over 65 years was 14.21%. Since 2018, Pudong New Area has entered the super-aging society with a proportion of 20.81% (Supplementary Figure 2A). Furthermore, the proportions of individuals aged 65–79 years and 80+ years showed significant upward trends during 1995–2018 (Supplementary Figure 2B). It is estimated that the elderly aged above 65 years in China will reach 322 million by 2050, accounting for one fifth of the total population (25). Thus, the burden of neurological disorders was concentrated in the elderly aged over 65 years, and the mortality and YLL varied among different age groups. It is necessary to take into account the age differences when analyzing the disease characteristics and intervention strategies for patients with neurological disorders.

In most cases, the etiology of neurological disorders is likely to be complex and involve an interplay between demographic and non-demographic factors. Decomposition method has been utilized to determine the relative contributions of exposures to differences in mortality rates among different groups of people (12, 26). In this study, we employed the decomposition method to quantitatively identify the contributions of demographic or non-demographic factors to the changing trend of mortality rates due to neurological disorders. Our results showed that the most important determinant of the increased rates in CMR during 1995–2018 was the demographic factor. In addition, the CMR varied among different age groups and CMR increased gradually with aging. Thus, aging is the main demographic factor associated with the increased rates in CMR of neurological disorders during the study period.

This study has major strengths including a large population size (3.0 million) and a relative long time span (24 years). However, there are several limitations to be acknowledged. First, the data regarding lifestyle, disease history, histological types, and medical care were unavailable in this study, so it was unable to identify the contributions of each risk factor that may result in changes in the mortality of neurological disorders. Second, as a descriptive study with its inherent disadvantages, it is difficult to confirm the causal association between socioeconomic status and cause-specific mortality. Although socioeconomic status affected the entire population, the extent of socioeconomic status in Pudong New Area could not be measured using indicators, resulting in a failure to quantify the associations. Third, although mortality registration system is of high quality, Pudong New Area is a district in Shanghai, and the representativeness of this study needs to be further discussed in the future. Fourth, as this study covered a 24-year period, data for 1995–2001 and 2002–2018 were coded based on ICD-9 and ICD-10, respectively. Potential differential misclassification of the causes of death may be introduced due to real-time adjudication and multiple ICD coding systems.

In summary, this study identified significantly increasing trends in mortality and burden of neurological disorders from 1995 to 2018 in Pudong New Area, a district that can represent typical rapid urbanization areas in developing world. The demographic factors, particularly aging, might be related to an increase in the mortality of neurological disorders. This population-based study can contribute to a better understanding of the particularities of neurological diseases at older ages and help in designing future preventive strategies in China and other similar cities around the world.

## Data Availability Statement

Data are available from the corresponding authors upon reasonable request and with permission of Center for Disease Control and Prevention of the Pudong New Area, Shanghai, China.

## Author Contributions

ZL, XL, and YD drafted the manuscript. XL and ZL participated in the collection, analysis, and interpretation of data. YD, YC, XX, and KL contributed to data collection and suggestion for analysis. XL, YZ, and YD conceived the study, participated in its design and coordination, and critically revised the manuscript. All authors read and approved the final version of the manuscript.

## Conflict of Interest

The authors declare that the research was conducted in the absence of any commercial or financial relationships that could be construed as a potential conflict of interest.
