# Supplementary material for: Years of Life Lost Due to Premature Death and Their Trends in People With Selected Neurological Disorders in Shanghai, China, 1995–2018: A Population-Based Study
Source: Front Neurol. 2021 Mar 5;12:625042. doi: 10.3389/fneur.2021.625042 (PMC7973274; doi:10.3389/fneur.2021.625042)
Supplement: Supplementary file 1 [file Data_Sheet_1.docx]

***Supplementary Materials***

**Years of life lost due to premature death and their trends in people with selected neurological disorders in Shanghai, China, 1995-2018: A population-based study**

**Zheng Luo, Huihui Lv, Yichen Chen, Xiaoyun Xu, Kangyong Liu, Xiaopan Li^*^, Yang Deng^*^ and Yi Zhou^*^**

**^*^ *Correspondence:*** *Xiaopan Li: xiaopanli0224@126.com.*

*Yang Deng: dengyang3417@126.com.*

*Yi Zhou: yzhou@pdcdc.sh.cn.*


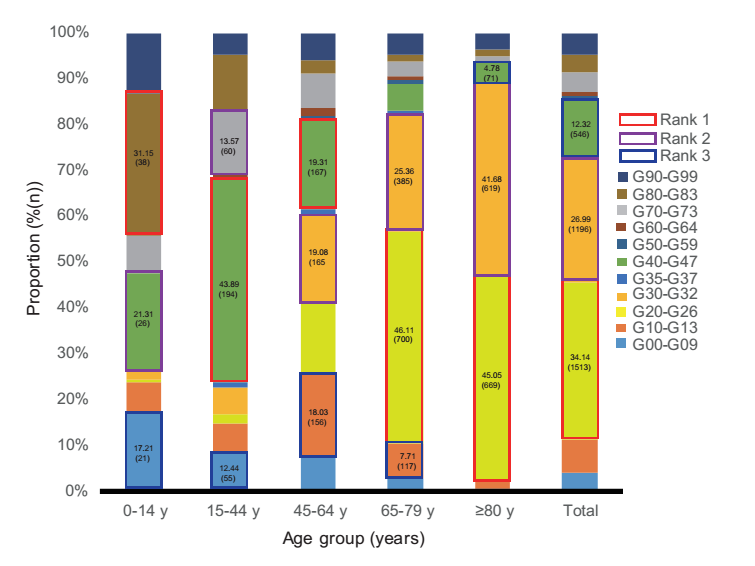
**Supplementary Figure 1** | The top tree death causes of selected neurological disorders among residents at different age groups in in Pudong New Area, Shanghai, China, 1995-2018. Abbreviations: G00-G09, inflammatory diseases of the central nervous system; G10-G13, systemic atrophies primarily affecting the central nervous system; G20-G26, extrapyramidal and movement disorders; G30-G32, other degenerative diseases of the nervous system; G35-G37, demyelinating diseases of the central nervous system; G40-G47, episodic and paroxysmal disorders; G50-G59, nerve, nerve root and nerve plexus disorders; G60-G64, polyneuropathies and other disorders of the peripheral nervous system; G70-G73, diseases of nerve junction and muscle; G80-G83, cerebral palsy and other paralytic syndromes; G90-G99, other disorders of the nervous system.


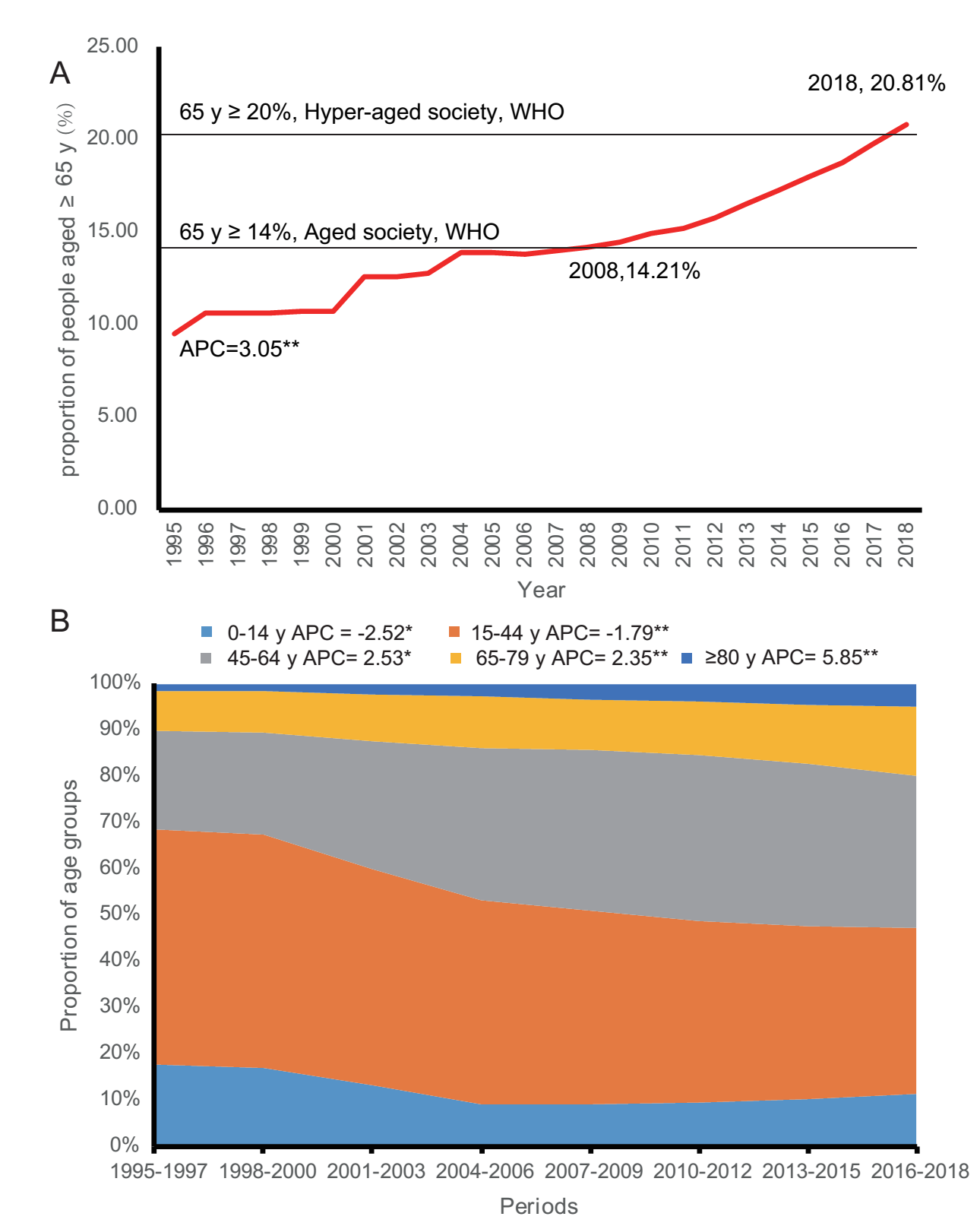
**Supplementary Figure 2** | Age composition of the population in Pudong New Area, Shanghai, China, 1995-2018. A: the proportion of people aged over 65 years; B: the trends in proportion of each age group from 1995 to 2018.

**Supplementary Table 1** | The increased rates caused by demographic and non-demographic factors and their contribution rates during the period from 1998 to 2018 compared with the CMR during 1995-1997

| **Characteristic** | **CMR of based period (/10^5^)** | **CMR of the other period (/10^5^)** | **D-value of CMR (/10^5^)** | **Impact of demographic factors** | |  | **Impact of non-demographic factors** | |
| --- | --- | --- | --- | --- | --- | --- | --- | --- |
|  |  |  |  | **Increased value (/10^5^)** | **Contribution rate (%)** |  | **Increased value (/10^5^)** | **Contribution rate (%)** |
| **1998-2000 vs.1995-1997** |  |  |  |  |  |  |  |  |
| Male | 4.88 | 3.96 | -0.92 | 0.12 | 189.38 |  | -1.04 | -89.38 |
| Female | 3.49 | 3.85 | 0.35 | 0.06 | 17.93 |  | 0.29 | 82.07 |
| Total | 4.18 | 3.91 | -0.28 | 0.09 | 180.46 |  | -0.37 | -80.46 |
| **2001-2003 vs.1995-1997** |  |  |  |  |  |  |  |  |
| Male | 4.88 | 5.50 | 0.62 | 0.85 | 121.64 |  | -0.24 | -21.64 |
| Female | 3.49 | 4.28 | 0.78 | 0.57 | 72.57 |  | 0.21 | 27.43 |
| Total | 4.18 | 4.89 | 0.70 | 0.67 | 95.68 |  | 0.03 | 4.32 |
| **2004-2006 vs.1995-1997** |  |  |  |  |  |  |  |  |
| Male | 4.88 | 6.65 | 1.77 | 1.67 | 94.60 |  | 0.10 | 5.40 |
| Female | 3.49 | 5.03 | 1.53 | 1.09 | 70.84 |  | 0.45 | 29.16 |
| Total | 4.18 | 5.84 | 1.66 | 1.32 | 79.48 |  | 0.34 | 20.52 |
| **2007-2009 vs.1995-1997** |  |  |  |  |  |  |  |  |
| Male | 4.88 | 8.85 | 3.97 | 2.46 | 62.04 |  | 1.51 | 37.96 |
| Female | 3.49 | 5.98 | 2.49 | 1.46 | 58.66 |  | 1.03 | 41.34 |
| Total | 4.18 | 7.42 | 3.23 | 1.87 | 57.85 |  | 1.36 | 42.15 |
| **2010-2012 vs.1995-1997** |  |  |  |  |  |  |  |  |
| Male | 4.88 | 8.25 | 3.37 | 2.69 | 79.98 |  | 0.67 | 20.02 |
| Female | 3.49 | 7.40 | 3.90 | 2.05 | 52.52 |  | 1.85 | 47.48 |
| Total | 4.18 | 7.82 | 3.64 | 2.28 | 62.74 |  | 1.36 | 37.26 |
| **2013-2015 vs.1995-1997** |  |  |  |  |  |  |  |  |
| Male | 4.88 | 9.92 | 5.04 | 3.59 | 71.27 |  | 1.45 | 28.73 |
| Female | 3.49 | 9.27 | 5.77 | 2.80 | 48.41 |  | 2.98 | 51.59 |
| Total | 4.18 | 9.60 | 5.41 | 3.08 | 56.97 |  | 2.33 | 43.03 |
| **2016-2018 vs.1995-1997** |  |  |  |  |  |  |  |  |
| Male | 4.88 | 12.20 | 7.32 | 4.61 | 62.96 |  | 2.71 | 37.04 |
| Female | 3.49 | 10.77 | 7.27 | 3.51 | 48.24 |  | 3.76 | 51.76 |
| Total | 4.18 | 11.48 | 7.30 | 3.91 | 53.64 |  | 3.38 | 46.36 |

CMR, crude mortality rate; D-value, difference value.
